# Reduction of the vertical vestibular-ocular reflex in military aircraft pilots exposed to tactical, high-performance flight

**DOI:** 10.3389/fneur.2023.949227

**Published:** 2023-06-09

**Authors:** Giovanni Bertolini, Alberto Pagnamenta, Andres Kunz, Aleardo Del Torso, Denis Bron

**Affiliations:** ^1^Aeromedical Center (AeMC), Swiss Air Forces, Dübendorf, Switzerland; ^2^Institute of Optometry, School of Engineering, University of Applied Sciences and Arts Northwestern Switzerland (FHNW), Olten, Switzerland; ^3^Department of Neurology, University Hospital of Zürich, Zürich, Switzerland; ^4^Clinical Trial Unit, Ente Ospedaliero Cantonale, Lugano, Switzerland; ^5^Department of Intensive Care Medicine, Ente Ospedaliero Cantonale, Lugano, Switzerland; ^6^Division of Pneumology, University Hospital of Geneva, Geneva, Switzerland; ^7^Otolaryngology Unit, Centromedico PDS Medical, Bellinzona, Switzerland

**Keywords:** vestibular-ocular reflex (VOR), aviation and aerospace, adaptation, video head impulse test (vHIT), supersonic flight, vertical semicircular canals, high-g flight

## Abstract

**Background:**

Exposure to high-performance flight stresses the vestibular system and may lead to adaptive changes in the vestibular responses of pilots. We investigated the vestibular-ocular reflex of pilots with different histories of flight exposure both with respect to hours of flight and flight conditions (tactical, high-performance vs. non-high-performance) to evaluate if and how adaptative changes are observable.

**Methods:**

We evaluated the vestibular-ocular reflex of aircraft pilots using the video Head Impulse Test. In study 1, we assessed three groups of military pilots: Group 1 had 68 pilots with few hours of flight experience (<300 h) in non-high-performance flight conditions; Group 2 had 15 pilots with many hours of flight (>3,000 h) and regularly flying tactical, high-performance flight conditions; Group 3 had eight pilots with many hours of flight (>3,000 h) but not exposed to tactical, high-performance flight conditions. In study 2, four trainee pilots were followed up and tested three times over a 4-year period: (1) <300 h of flight on civil aircraft; (2) shortly after exposure to aerobatic training and with <2,000 h of overall flight; and (3) after training on tactical, high-performance aircraft (F/A 18) and for more than 2,000 h of flight.

**Results:**

Study 1: Pilots of tactical, high-performance aircrafts (Group 2) had significantly lower gain values (*p* < 0.05) as compared to Groups 1 and 3, selectively for the vertical semicircular canals. They also had a statistically (*p* = 0.022) higher proportion (0.53) of pathological values in at least one vertical semicircular canal as compared to the other groups. Study 2: A statistically significant (*p* < 0.05) decrease in the rVOR gains of all vertical semicircular canals, but not of the horizontal canals, was observed. Two pilots had a pathological value in at least one vertical semicircular canal in the third test.

**Discussion:**

The results evidence a decrease in the gain of the vestibular-ocular reflex as measured with the video head impulse test for the vertical canals. This decrease appears to be associated with the exposure to tactical, high-performance flight rather than with the overall flight experience.

## Introduction

Continuous integration and interpretation of multi-sensory motion cues allow human beings to derive a percept of orientation and self-motion and to maintain it coherently against sensory noise and transient perturbations. The process of extracting self-motion cues from visual, vestibular, and proprioceptive systems is optimized for our natural self-propelled motion (walking, running, and jumping) and may be challenged by combinations of inputs that are captured by our sensory system in an unnatural motion environment, such as flying an airplane (1).

A major risk during flight originates from the misinterpretation of the combination of motion cues and/or from the sensorineural conflicts deriving from incorrect processing. Both conditions can cause a number of aberrant responses, ranging from the inappropriate triggering of motor reflexes to cognitive or systemic reactions (i.e., spatial disorientation, motion sickness, and Sopite syndrome) (1). Altogether they may create discomfort, impact performance (e.g., from improper eye targeting to impaired decision-making), and induce a misperception (with potential risks of incorrect, possibly lethal, maneuvers) compromising the quality and safety of flights.

Advances in aerospace technology progressively allow higher speed, higher acceleration, and a higher rate of turn. This may further increase the abovementioned risks, triggering combinations of motion cues further away from the optimal working range of human self-motion perception. If and how prolonged exposure may impair functionally relevant reflexes is a critical open question for aviation medicine.

The rotational-vestibulo-ocular reflex (rVOR) stabilizes images on the retina during head rotations by counterrotating the eyes (2). The rVOR is triggered by the stimulation of ampullary receptors in the semicircular canals in the inner ear (sensitive to angular accelerations of the head). The signal is transmitted by a three neurons arc to the eyes' muscles. It is a very fast reflex taking 5–10 milliseconds from the sensory stimulation to extraocular muscle activation, providing the earliest gaze stabilization following head movements.

During natural self-propelled motion, the rVOR works in combination with other systems (e.g., optokinetic, smooth pursuit) to provide adequate stabilization of the visual field during head movements.

However, during flight, head movements do not occur in natural conditions. The cockpit pilot needs to perform numerous head-eye movements to look at both internal (cockpit) and external targets. During these movements, the vestibular system also captures stimuli resulting from airplane motion and artifacts stemming from the relative motion of the head in a moving environment (1). The semicircular canals in the inner ear cannot distinguish between angular accelerations from active head motion (e.g., head tilt) and the head motion that are imposed by the vehicle motion (e.g., if the plane rolls to the left, the head rolls with it). As the vehicle motion is not in the range of natural head motion to which the vestibular system responds correctly, the resulting combinations may trigger aberrant, inappropriate eye movements.

Gaze stability is, however, essential for pilots. The rVOR is plastic and adapts to repetitive or prolonged stimuli. Bidirectional or unidirectional response decrease has been demonstrated in numerous studies employing adaptation protocols (3, 4). Spontaneous adaptation has also been reported in athletes experiencing intensive vestibular stimulation (figure skaters, ballet dancers, and gymnasts) (5). It is thus expected that repeated exposure to motion stimuli experienced in flight will drive adaptive changes in the vestibular processing of pilots, which may be identifiable by changes in the rVOR. The impact on the health of flight exposure can be assessed by quantifying how pilots adjust to these stimuli, allowing them to monitor flight risks.

Previous studies on the adaptation of rVOR in pilots showed conflicting results. Higher rVOR gains were reported in pilots and in-flight students as compared to control subjects using a rotating chair (frequency range of 0.01–0.32 Hz) (6, 7). The increase in rVOR gain in student pilots was reported to occur after the initial flight training (6). Two studies using rotatory chairs (one with oscillation in the frequency range of 0.01–0.32 Hz and the other with velocity steps), however, reported no differences in the rVOR between pilots and non-pilots (8, 9). The only previous study that tested the rVOR in aircraft pilots using head impulses (Head-impulse Test, HIT) observed lower gain values for the vertical canals but with significance limited to the left posterior canal. The study, however, only measured 14 pilots, of which only six of them had a large number of hours in flight (10).

These discrepancies may be related to the differences in the motion stimuli experienced by pilots (9, 10). The magnitude of angular and linear accelerations experienced by a fighter pilot, an aerobatic pilot, or a civil pilot varies considerably. Similarly, the time on instrument flight compared to the time on visual flight varies, possibly affecting the adaptation drive (e.g., due to the different coherence between visual and vestibular cues or the gaze stabilization demand).

In choosing how to test the rVOR, we consider that rVOR adaptation is known to be frequency dependent (11). Rotary chairs as those used in the previous studies measure the VOR responses between 0.01 Hz and 0.64 Hz (11). While the rVOR responses at these frequencies allow us to appreciate the contribution of central processing of semicircular canal signals (including velocity storage, which is considered relevant for motion perception and motion sickness) (12, 13), the majority of natural head movements range between 0.5 Hz and 5 Hz (14). It is thus possible that the studies of rVOR responses to 0.01–0.64 Hz have missed adaptations to higher frequencies. The pilot inside the cockpit must indeed perform many rapid movements of the head to keep the instrumental data under control (e.g., position, direction, speed, and altitude) without losing sight of the surrounding environment, especially when tracking a visual target. The rapid movements of the head are thus most likely within the upper part of the natural frequency range of the rVOR.

Under the hypothesis that the adaptive demand to the rVOR in pilots of tactical, high-performance aircraft (high-performance aircraft is defined as an aircraft with more than 200 HP) is related to maintaining gaze stability while exposed to aberrant vestibular stimulations (e.g., Coriolis/cross-coupling, high rate of turn or sustained gravito-inertia tilt), we decided to measure rVOR using the HIT.

The head impulse test, described for the first time in 1988 by Halmagyi and Curthoys (15), is one of the key tests for the clinical evaluation of vestibular function. It is the only “bedside” test that allows for the identification of the hypofunctional side within a unilateral peripheral vestibulopathy (unilateral vestibular loss, UVL) (15). In the HIT test, the examiner holds the patient's head with two hands. The patient must keep his gaze fixed on the tip of the examiner's nose, who will perform repetitive sudden passive rotations (known as “impulses”) of the head in one of the planes containing the functional pair of semicircular canals that are being tested [horizontal, LARP, left anterior/right posterior or RALP, right anterior/left posterior (16)]. With a normal rVOR, the patient should be able to constantly stare at the examiner's nose, and alternatively, the patient's gaze moves in the same direction as the head as eye movements do not perfectly compensate for head rotation. The video head impulse test (vHIT) is an objective indicator of the HIT (17). The patient wears goggles containing high-speed cameras (250 Hz) for videooculography (VOG) and accelerometers measuring head movement. The rVOR function is thus quantified in terms of gain, which is defined as the ratio between the position change of the eyes and the position change of the head occurring during the head impulse (17).

The two studies presented in this study aim to shed light on this topic. For the first time, we attempted to quantify how the vestibular adaptative changes occurring in airplane pilots depend both on the hours of flight and the kind of exposure, mixing retrospective and perspective approaches. The retrospective study compares pilot trainees with a few hours of flight with experienced pilots with and without exposure to tactical, high-performance flight. The other study followed four jet-fighter pilots during training until and including their initial exposure to tactical, high-performance flight. We hypothesized that a stronger adaptation of rVOR to the sensory conflicts induced by head motions in flight will be associated with tactical, high-performance flight. Specifically, considering that tactical, high-performance aircraft aviators report a significantly higher incidence of spatial disorientation, we speculated that an inhibited vestibular response will be observed (18–20).

In contrast to the rotatory chair test used in the previous study, vHIT examines rVOR in the upper range of frequency (2–5 Hz). It is therefore expected that our results will be complementary to the current literature.

## Methods

The current study reports the results of two studies. In study 1, the rVOR of military pilots with different histories of exposure to flight conditions was compared. In study 2, the rVOR of pilot trainees was recorded at different time points during 4 years of training.

### Subjects

In study 1, a total of 90 male military pilots active in the Swiss Air Force between 2014 and 2017 were included. The pilots were divided into three groups based on the kind of exposure to flight conditions. Group 1 (trainee group) included 68 military pilots (20–27 years of age) in the initial training phase (10–300 flight hours with non-high-performance/non-aerobatic conditions); group 2 (high-performance group) included 15 veteran military pilots (26–47 years) active on tactical, high-performance aircraft (F/A 18; >3,000 h flight hours); and group 3 (non-high-performance group) included seven veteran military pilots (30–53 years) that were never active on tactical, high-performance aircraft (>3,000 h flight hours on PC/7 and other non-high-performance aircraft).

In study 2, four trainees were followed up during 4 years of training. The vHIT was performed three times: (1) after a few hours of flight (quantified approximately 30–300 h of flight, civil aircraft); (2) at an intermediate phase following exposure to aerobatic flight training (300–2,000 h of flight; including aerobatic flight and training sessions in a centrifuge); (3) after starting training on F/A 18 (>2,000 h of flight; only military flight with fighter aircrafts).

All subjects volunteered for the study and signed informed consent. This study was approved by the Ethics Committee of the University of Zurich (Gesuch Basec Nr. 2016-01230) in Switzerland.

### Set-up and test procedure

All tests were performed using the Otometrics OTOsuite Vestibular [v1.2.0, 2012 and v4.0, 2016—the software has been updated from to Otometrics OTOsuite Vestibular 4.00 Build 1286 (21) between the second and the third test performed on the pilots of study (2)].

All tests were performed by the same examiner. During the examination, the pilots were seated on a chair positioned at ~1.20 m from a wall where a visual target is located. This allows the pilot's eyes to be 1 m from the target (optimal distance as it ensures the non-convergence of the eyes, which would lead to an increase in the measured rVOR gain) (17). The software guided the operator to orient the head of the pilot according to the plane to be tested (lateral / HSCCs; LARP; RALP). Eye movement data were calibrated using the standard two targets procedure provided by the software. After the calibration, the examiner asked the pilot to fixate the target on the wall and then imposed rapid, unpredictable rotations on the patient's head with a small amplitude. To test the vertical canals (LARP and RALP), the pilot's head was rotated by 35–45° with respect to the body so that the target lay in the plane of the vertical canals. In this position, compensation for the stimulation of the vertical channels requires an almost exclusive vertical eye movement to keep the target on the fovea.

### Eye movement analysis

Analysis of eye movements was performed using the automatic routine of the software from GN Optometric (21). The software selects the head movements in the predefined range, excluding those that could lead to diagnostic errors (e.g., the error of the rebound in the execution of an outward movement, impulses at inadequate speeds, or not lying along the plane of the studied channels) and remove artifacts due to blinks. The output consists of VOR gains (area under eye velocity curve/area under head velocity curve) for each of the tested canals. To compute the VOR gains, the software requires a minimum of 20 “good” impulses in each direction.

### Statistical analysis

Data are summarized as mean with standard deviation (SD) or as median with the 25th and 75th percentile, as appropriate. Comparisons among the three different pilot groups in Study 1 were performed with a one-way analysis of variance (ANOVA) or with the Kruskal–Wallis test as appropriate (after checking the assumptions of the ANOVA). When the *F*-ratio of the ANOVA reached a critical level (corresponding to a *p* < 0.05), a *post hoc* analysis with Bonferroni correction was employed. By using the statistically significant Kruskal–Wallis test, a *post hoc* analysis with the Mann–Whitney test, taking into account the multiple testing procedure, was used. In study 2, due to the non-independency of the data, non-parametric repeated measures of ANOVA (Friedman test) were used to compare all variables at different time points. In study 2, a *post hoc* analysis was not foreseen because our primary interest was on trend in time and not on the single time point results. In addition, to test whether the proportion of pathological values was comparable between groups, Fisher's exact test was used.

All tests were performed two-sided, and a *p*-value of < 0.05 was considered to be statistically significant. All analyses were performed with STATA 15 (StataCorp LP, College Station, TX, USA).

## Results

Table 1 shows the rVOR gain found in study 1 per each semicircular canal in each group and reports the *p*-values of statistical comparisons.

**Table 1:** VOR gain reflexes for the six semicircular canals (Group 1 × Group 2 and 3).

	**Group 1 (trainee group) *n* = 68**	**Group 2 (high-performance group) *n* = 15**	**Group 3 (non-high-performance group) *n* = 8**
GLL^#^	0.901 ± 0.057	0.846 ± 0.040^¶^	0.87 ± 0.041
GRL^#^	0.954 ± 0.057	0.962 ± 0.043	0.98 ± 0.059
GLA^&^	0.931 ± 0.232	0.735 ± 0.106^¶^	1.15 ± 0.119^*^^§^
GRP^#^	0.957 ± 0.288	0.736 ± 0.064^*^	1.15 ± 0.160^§^
GLP^&^	1.014 ± 0.183	0.846 ± 0.061^¶^	1.08 ± 0.054^§^
GRA^&^	1.072 ± 0.163	0.876 ± 0.267^*^	1.07 ± 0.165

* *p*-value < 0.05 compared with group 1;

¶ *p*-value < 0.01 compared with group 1;

§ *p*-value < 0.01 compared with group 2. Data are presented as mean ± standard deviation. GLL, gain left lateral canal; GRL, gain right lateral canal, GLA, gain left anterior canal; GLP, gain left posterior canal; GRA, gain right anterior canal; and GRP, gain right posterior canal. Group 1: military pilots in initial training flight; Group 2: military pilots exposed to accelerator stresses on tactical, high-performance aircraft; and Group 3: experienced military pilots not regularly exposed to the demands of tactical, high-performance aircrafts.

# The comparison among groups was performed with one-way ANOVA.

& The comparison among groups was performed with the Kruskal–Wallis test. In both situations, by significant *p*-value, pairwise *post-hoc* analysis with *p*-value correction for multiple comparisons was performed.

VOR gains in group 2 (high-performance group) were significantly lower than in group 1 (trainee group) for all semicircular canals except for the right lateral one and significantly lower than in group 3 (non-high-performance group) for the left anterior, right posterior, and left posterior region. A significant difference was also found between groups 1 (trainee group) and 3 (non-high-performance group) with the first having a lower left anterior gain.

As the majority of mean values reported in Table 1 are above the threshold for pathological findings (as specified by the manufacturer, 0.8 for horizontal and 0.7 for vertical canals), the reported difference may be considered incidental as they stay below the detection threshold of the instrument. To investigate the clinically relevant results, we compared the incidence of individual pathological findings across the groups.

No pilot had pathological gains in the horizontal canals, but pathological values were found for the vertical canals. In group 1 (trainee group), 12/68 (18%) pilots had at least one gain lower than 0.7 in the vertical canal (range: 0.69–0.47, median 0.62; 4/68 pilots with pathological gain in more than one canal; 11 pilots with pathological gain in the posterior right, four in the anterior left; one in the anterior left). In group 2 (high-performance group), 8/15 (53%) pilots had pathological gain values in the vertical canals (range: 0.69–0.13, median 0.66; 1/15 pilots with pathological gain in more than one canal; two in the anterior right, four in the posterior right, and three in the anterior left). In group 3 (non-high-performance group), no pathological values were found.

Fisher's exact test confirmed that the proportions of pathological gain observed among groups were different. Specifically, the proportion in group 2 (high-performance group) was statistically higher than in group 1 (trainee group) (*p*-value = 0.007) and in group 3 (non-high-performance group) (*p*-value = 0.022).

**Figure 1:**
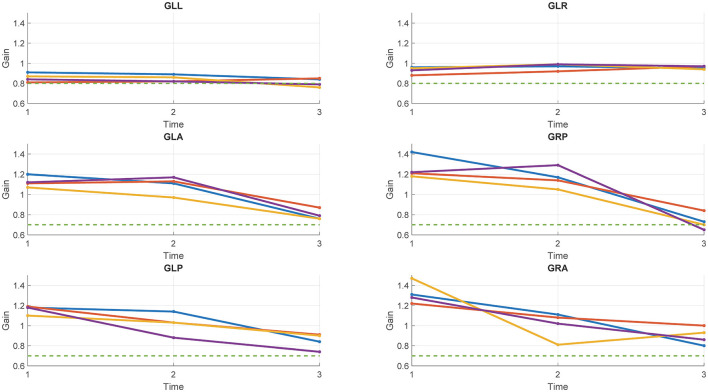
Gains of the six semicircular canals of the four pilots tested in study 2 at each of the three time points. Each line represents a pilot. Green dotted line: Threshold for the pathological outcome of the vHIT (0.8 for the lateral canals; 0.7 for the vertical canals).

**Figure 2:**
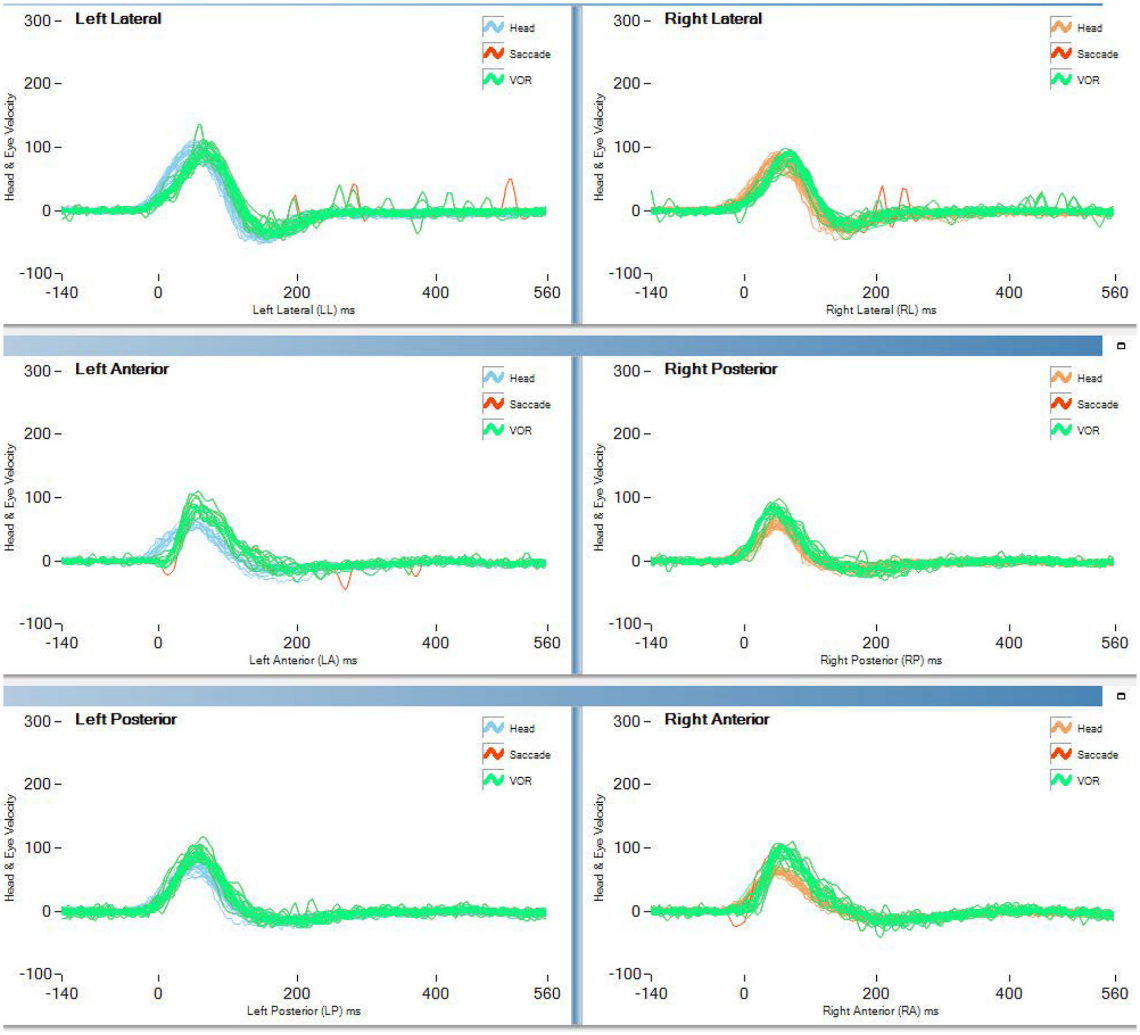
vHIT traces of the first test (<300 h no aerobatic, no F/A 18) of pilot 1 of study 2 (blue line in Figure 1).

**Figure 3:**
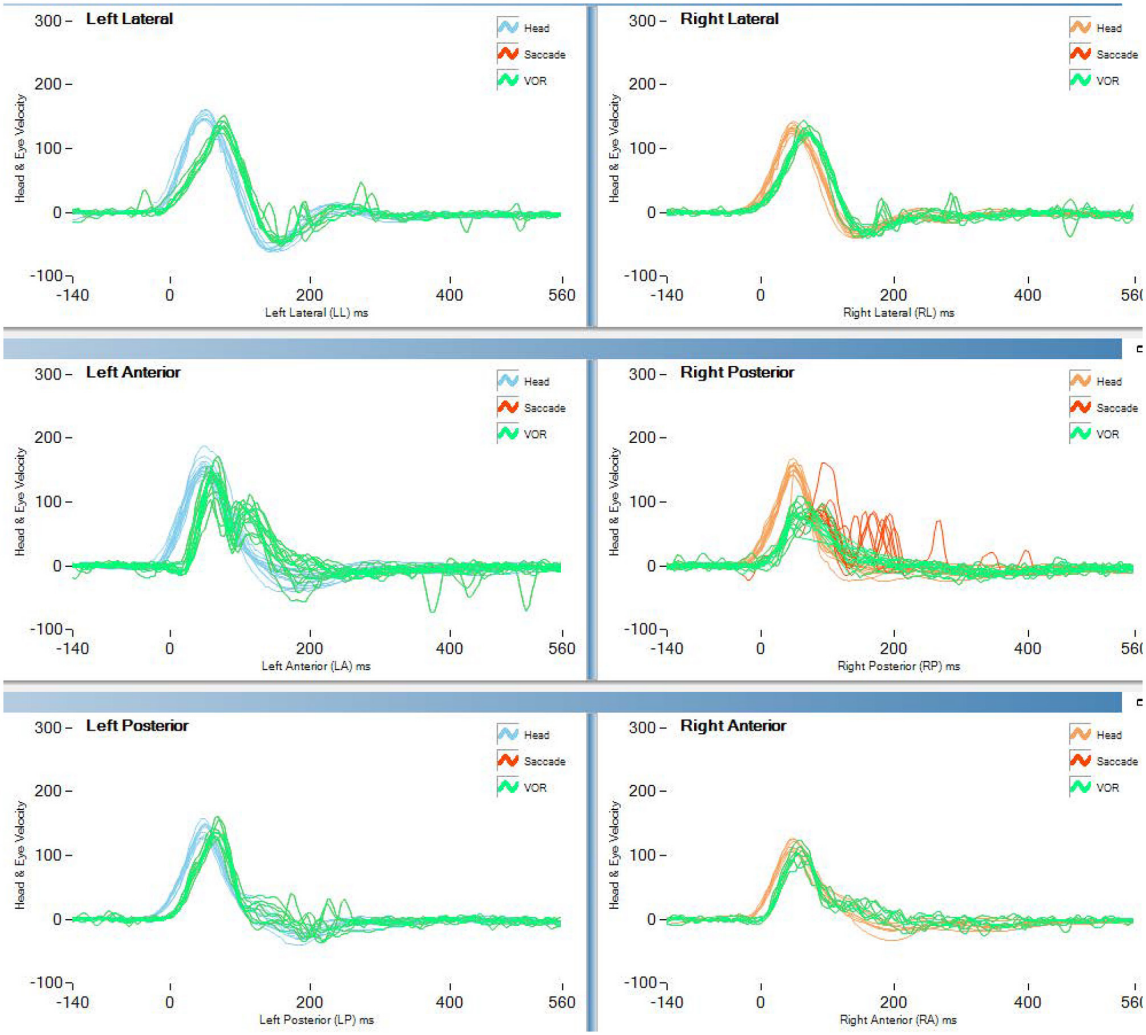
vHIT traces of the last test (>2,000 h aerobatic F/A 18) of pilot 1 of study 2 (blue line in Figure 1). Both covert and overt saccades are visible in the vertical canal traces.

Table 2 and Figure 1 report the results of Study 2. The analysis revealed a statistically significant decrease in the rVOR gains of the vertical semicircular canals but not of the horizontal canals. Two pilots had a pathological test outcome in one vertical semicircular canal (both in RP) and one lateral canal (LL) after training on tactical, high-performance aircraft. This emerges also as an observation of the eye movement traces. Comparing the eye movement responses recorded during the first visit to impulses testing the RP semicircular canals (Figure 2—RP panel) with the matching responses recorded during the third visit (Figure 3, RP panel), the occurrence of corrective saccades (including covert saccades) can be observed. The individual VHIT traces of all semicircular canals of all pilots at each of the three time points are reported in Appendix 1.

**Table 2:** VOR gain reflexes for the six semicircular canals of four pilots values recorded in three different training periods.

**Flight experience**	**Time 1** **< 300 h; no aerobatic; no F/A 18**	**Time 2** **>300 & < 2,000 h; aerobatic; no F/A 18**	**Time 3** **>2,000 h; aerobatic; F/A 18**	***p*-value**
GLL	0.855 (0.825; 0.89)	0.84 (0.82; 0.875)	0.815 (0.775; 0.845)	0.105
GRL	0.94 (0.905; 0.955)	0.98 (0.945; 0.99)	0.96 (0.945; 0.97)	0.257
GLA	1.115 (1.09; 1.16)	1.12 (1.04; 1.15)	0.775 (0.76; 0.83)	0.038^*^
GRP	1.215 (1.195; 1.32)	1.155 (1.095; 1.23)	0.715 (0.675; 0.785)	0.038^*^
GLP	1.18 (1.14; 1.185)	1.03 (0.955; 1.085)	0.87 (0.79; 0.905)	0.013^*^
GRA	1.295 (1.25; 1.39)	1.05 (0.915; 1.095)	0.895 (0.83; 0.965)	0.038^*^

Data are presented with median and 25th and 75th percentiles. GLL, gain left lateral canal; GRL, gain right lateral canal; GLA, gain left anterior canal; GLP, gain left posterior canal; GRA, gain right anterior canal; GRP, gain right posterior canal. Time 1: initial training phase (only PC-7 flight); Time 2: intermediate training phase, after exposure to aerobatic flight with PC-7; Time 3: advanced training phase with regular exposure to tactical, high-performance flight with F/A 18. ^*^p < 0.05.

## Discussion

The combined results of the two studies presented in this study suggest that prolonged exposure to tactical, high-performance flight may lead to a reduction of vertical rVOR gain in response to rapid head movements (as tested with vHIT). While the reported average gains are all within the normative limits, the relevance and the potential impact of the finding are highlighted by the high proportion of pilots exposed to tactical, high-performance flight conditions tested in study 1 that presents pathological findings (>50%). The comparison between groups of pilots with more than 3,000 h of flight, with and without exposure to tactical, high-performance flight, suggests that is not simply the flight exposure that is associated with a decrease of rVOR gain but rather the exposure to specific flight conditions. The hypothesis of a relationship between the reduced vertical rVOR gain and the hours of tactical, high-performance flight is further corroborated by the small prospective observational study on four student pilots (study 2), where significant gain reductions were observed for all four vertical canals. In addition to the gain reduction, covert and overt saccades were observed in this group, especially after >2,000 h of flight and exposure to tactical, high-performance flight. The presence of corrective saccades provides strong support for our evidence of a change in gain.

Given the small size of the sample, we consider that attempting to provide an explanation for the occurrence of covert vs. overt saccades would be too speculative.

The results imply that the isolated lower gain reported in a previous study (10) for the vertical canals was not an incidental finding. Statistical significance may have not been achieved due to the limited number of pilots tested (14 in total). Furthermore, the study tested groups with quite heterogeneous flight experience (1,000–3,000 h of flight), and the kind of flight exposure was not differentiated.

Although the current study cannot exclude that significant gain decreases may be observed for the horizontal canal in a larger dataset, it is noteworthy that significant effects were found for vertical rVOR only. A selective adaptation may suggest that the conflicts between perceived angular and linear accelerations (e.g., as in Coriolis/cross-coupling stimuli) play an important role in the adaptive process. Vertical head movements during our normal motion imply a reorientation with respect to gravity, i.e., a change of the gravito-inertial vector sensed by the otolith organs. Accordingly, vertical rVOR gain has been shown to be modulated by concurrent otolith signals (22). Specifically, the vertical rVOR gain was enhanced when the otolith signal was compatible with the respective rotation in the vertical plane, a phenomenon not observed for horizontal rVOR (23), where only the rVOR time constant is affected. Head pitch and head roll in the cockpit may induce Coriolis/cross-coupling stimuli, with strong conflicts between sensed angular rotation and gravito-inertial acceleration (24). It can be speculated that repetitive exposure to stimuli causing suppression of vertical rVOR gain may lead to an overall decrease in the gain. This idea would also provide a possible explanation of why the significant decrease was found only in pilots flying in tactical, high-performance aircrafts, which are exposed to higher linear accelerations as compared to the other pilot tested and thus to stronger canal-otolith conflicts.

While the finding of a decreased gain may appear in contrast with the significant increase observed in earlier studies (6, 7), we believe that our study does not oppose but rather complements the previous findings. Head impulses employ brief, high-speed stimuli probing rVOR responses in the higher portion of the frequency range of natural head movements (2–5 Hz), while the rotatory chair probes rVOR at lower frequencies (0.1 Hz−0.64 Hz). Furthermore, vHIT mainly exploits the oligosynaptic pathways of the VOR that project from the semicircular canals to the nuclei of the extrinsic ocular muscles. These pathways provide the fastest gaze stabilization response.

It can be speculated that a selective gain reduction in these pathways may even be functionally useful for pilots who need to focus on instruments. The rVOR triggered by aircraft rotations is functional to stabilize vision for external targets, but it needs to be suppressed to fixate cockpit instruments, which have no relative motion with respect to the head. An rVOR with a lower gain may be easier to suppress even when the rate of turn is elevated (e.g., as in jet-fighter roll). The functional role of the observed reduction cannot however be clarified by the current study.

Rotatory chair tests investigate the entire rVOR response, including the effect of the polysynaptic pathways that involve the cerebellum (2). These pathways are supposedly serving a multi-sensory integration function through the velocity storage system, which is known to be adaptable (1). Specifically, lower VOR gains have been associated with adaptive responses to repeated sea exposures (25). Another study suggested that rVOR gain remains unaffected by adaptation to conflicting motion stimuli (26).

Combining our evidence with previous findings, an adaptation of the rVOR to the stimuli of flight thus appears as a complex process. In line with recent studies describing the frequency-dependent adaptation of the rVOR (11), it can be speculated that, depending on the exposure and the gaze stabilization demand (i.e., on the kind of in-flight exposure), a low-frequency gain of the rVOR may be increased, while high-frequency gain decreased, thus bringing our and previous results together.

As evidence suggests that both phenomena have been observed mostly in tactical, high-performance aircraft pilots, it is possible that strong multi-sensory conflicts are the drive of the adaptation. The stimuli that trigger the different adaptation processes are however still need to be determined, as well as whether these adaptations are independent or co-occurring.

We previously reported a case study of a pilot who suffered from air sickness. In this pilot, a desensitization protocol was associated with a decrement in the rVOR gain on both sides (27). While this suggests that adaptation of the polysynaptic and oligosynaptic pathways may co-occur, further studies assessing rVOR over the whole frequency spectrum using more than one diagnostic method on the same pilots are needed. Without a study consistently comparing rVOR using both the rotatory chair and vHIT in pilots with different flight exposure, it is indeed not possible to conclude that the rVOR adaptation is frequency dependent.

It can be speculated that an alternative, non-vestibular explanation for the finding presented in this study is a change in neck stiffness. Neck pain and injuries arising from strain or stiffness of the neck are commonly reported by aircraft pilots (28, 29), and it is also a known challenge in performing vHIT as the test requires producing a small, rapid rotation to the participant's head. The possibility that the geometrical nature of the stimuli (and thus of the rVOR response) may differ between a patient with a relaxed neck and one with a stiff neck has also been considered (17). While these considerations suggest neck stiffness as a possible candidate for explaining our results, it is unlikely that an increase in neck stiffness with tactical, high-performance flight exposure is the cause of the observed results. First, although neck stiffness limits the testing procedure of vHIT, possibly reducing head velocity or motion amplitude (30), we could not find a study providing evidence that a reduction of rVOR gain has been observed. Furthermore, the results found a lower gain for vertical canals only, as well as why neck stiffness may also affect vHIT head impulses testing horizontal canals. Finally, as neck problems are a well-known constraint for performing vHIT, an abnormal occurrence of neck stiffness would have been noticed by the operator performing the test.

In light of these considerations, we consider that the hypothesis of vestibular adaptation is more likely to explain the observed results. The question arises as to whether the VOR adaptation can interfere with the quality of flight, in particular, if it can interfere with gaze stabilization. The adaptation process that allows physically enduring the repeated neurosensory conflicts during a flight on military jets can potentially expose the pilot to errors of assessment on its targets. In light of this question, it will be useful to evaluate the repercussions of this functional lowering of the VOR, studying the values obtained using the head impulse testing device (HITD). The HITD appears to be a promising tool for detecting abnormal VOR performance while providing information on the functional performance of the rVOR (31). This evaluation will provide further information on the processes of adaptation of the human body to in-flight stresses to optimize training processes and reduce the risk for pilots.

## Data availability statement

The raw data supporting the conclusions of this article will be made available by the authors, without undue reservation.

## Ethics statement

The studies involving human participants were reviewed and approved by Kantonalen Ethikkommission Zürich. The patients/participants provided their written informed consent to participate in this study.

## Author contributions

GB co-wrote the manuscript, analyzed the data, critically interpret the results, and draw the conclusions. AP analyzed the data and critically revised the manuscript. AK supported the study design and helped drawing conclusion. AD conceived the study, co-wrote the manuscript, collected the data, and interpreted the result. DB co-conceived the study, critically revised the manuscript, and participated in drawing the conclusion.
