# Supplementary material for: Reduction of the vertical vestibular-ocular reflex in military aircraft pilots exposed to tactical, high-performance flight
Source: Front Neurol. 2023 Jun 9;14:949227. doi: 10.3389/fneur.2023.949227 (PMC10289026; doi:10.3389/fneur.2023.949227)
Supplement: Supplementary file 1 [file Data_Sheet_1.PDF]

APPENDIX 1

This Appendix presents the VHIT traces of all four pilots of study 2 at all 3 timepoints.

Pilot 1: Blue line in Figure 1 (Figure A1 und A3 are also presented in main text)

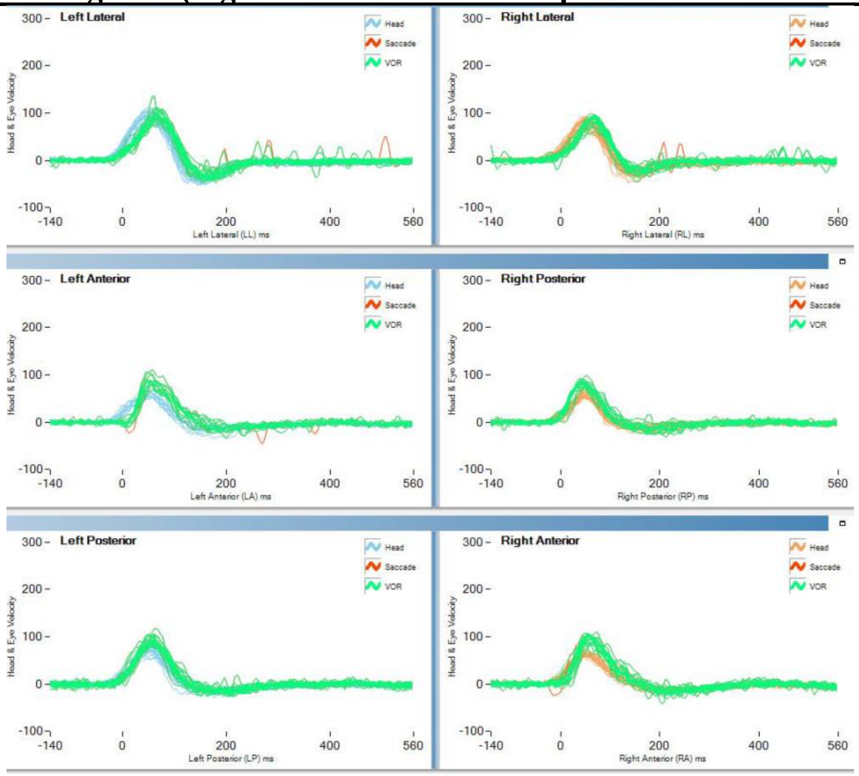

Figure A1: vHIT traces of the first test (<300h no aerobatic, no F/A 18) of pilot 1 of study 2

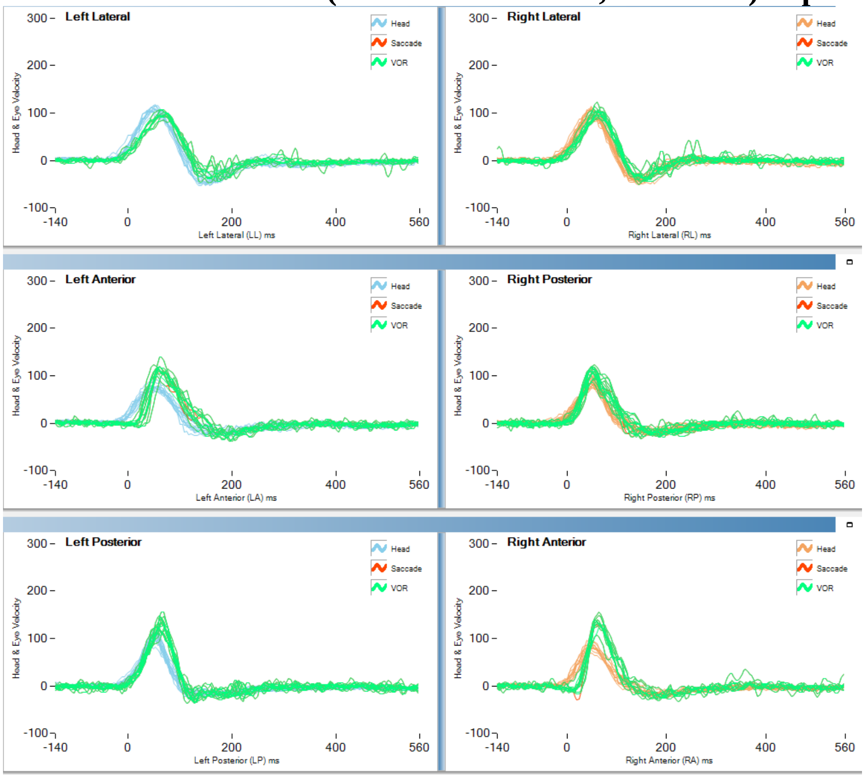

Figure A2: vHIT traces of the second test (>300h & <2000h, aerobatic, no F/A 18) of pilot 1 of study 2

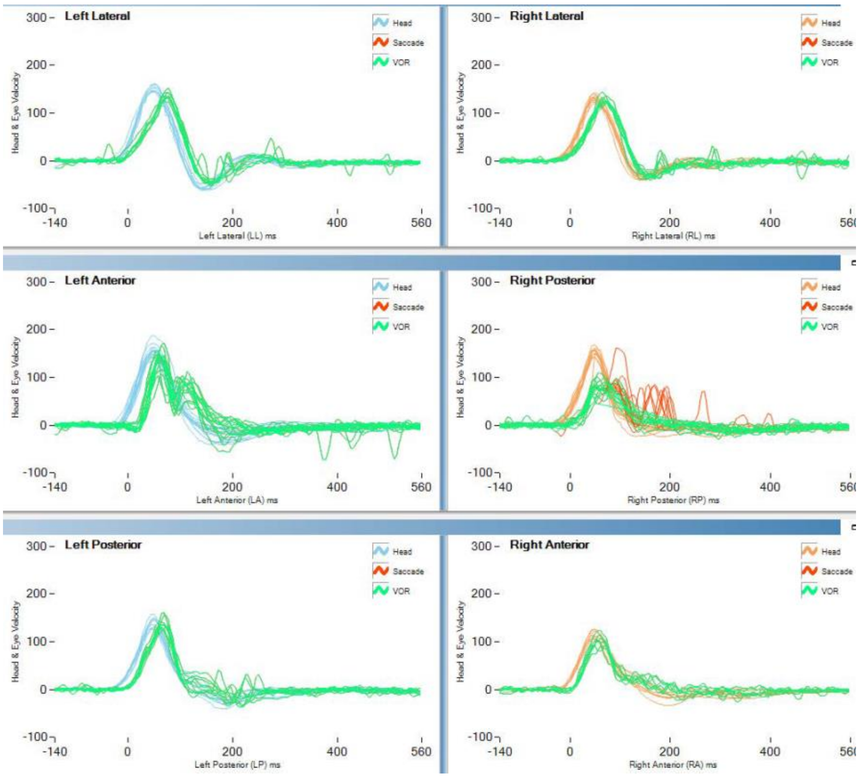

Figure A3: vHIT traces of the last test (>2000h aerobatic & F/A 18) of pilot 1 of study 2

<u>Pilot 2: Yellow line in Figure 1</u>

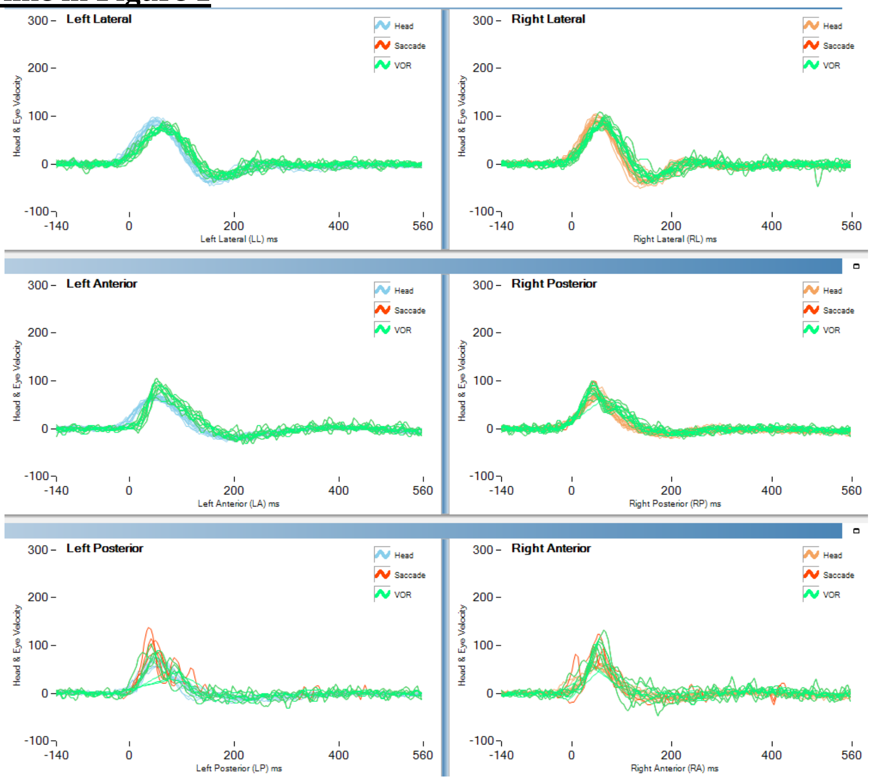

Figure A4: vHIT traces of the first test (<300h no aerobatic, no F/A 18) of pilot 2 study 2

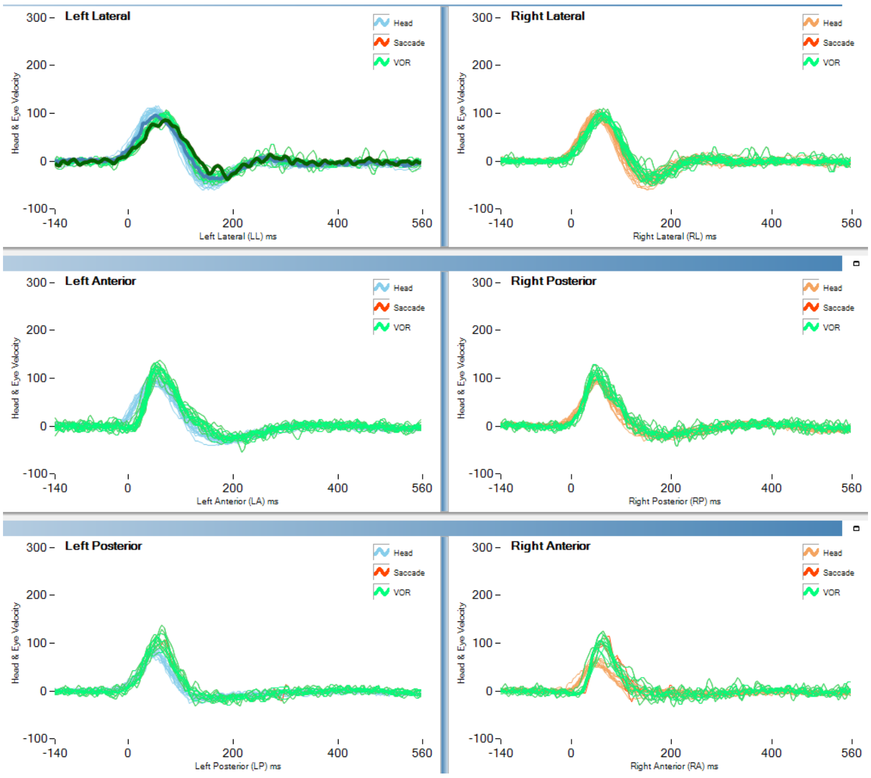

Figure A5: vHIT traces of the second test (>300h & <2000h, aerobatic, no F/A 18) of pilot 2 of study 2

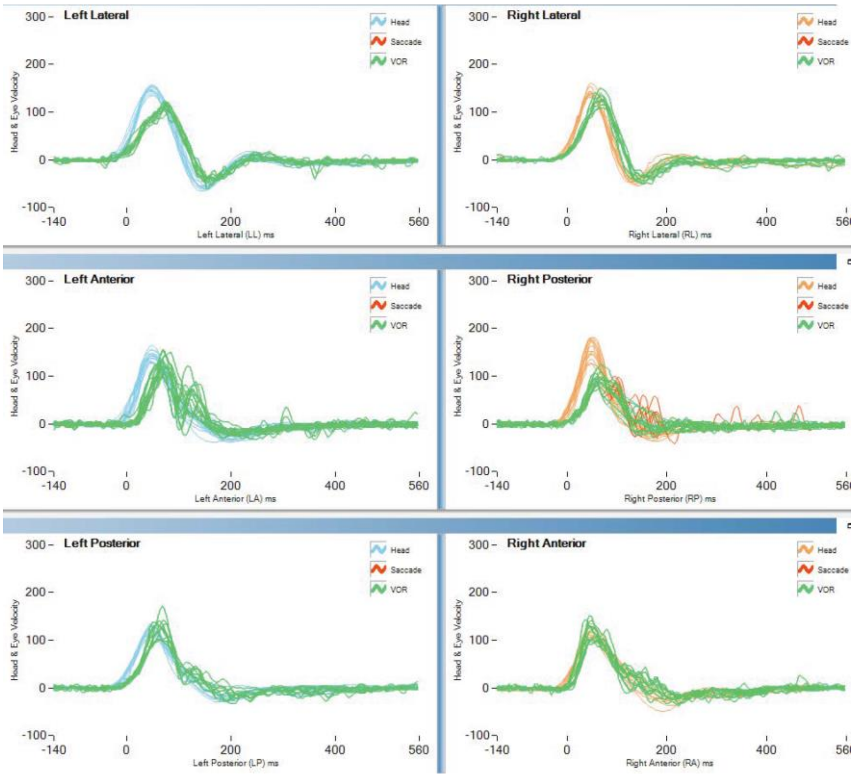

Figure A6: vHIT traces of the last test (>2000h, aerobatic & F/A 18) of pilot 2 of study 2

<u>**Pilot 3: Red line in Figure 1**</u>

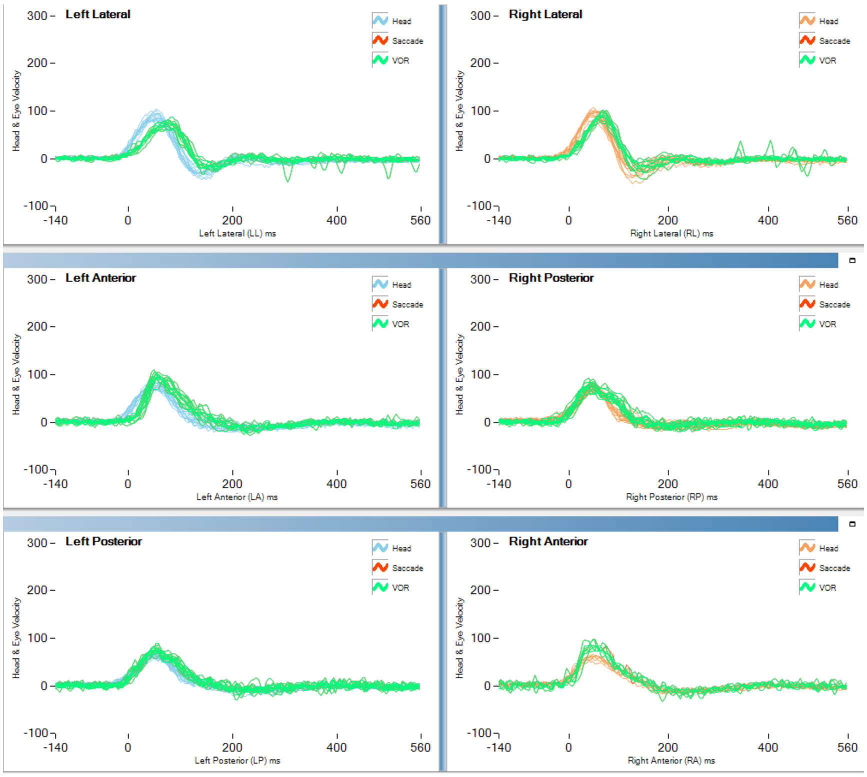

Figure A7: vHIT traces of the first test (<300h no aerobatic, no F/A 18) of pilot 3 of study 2

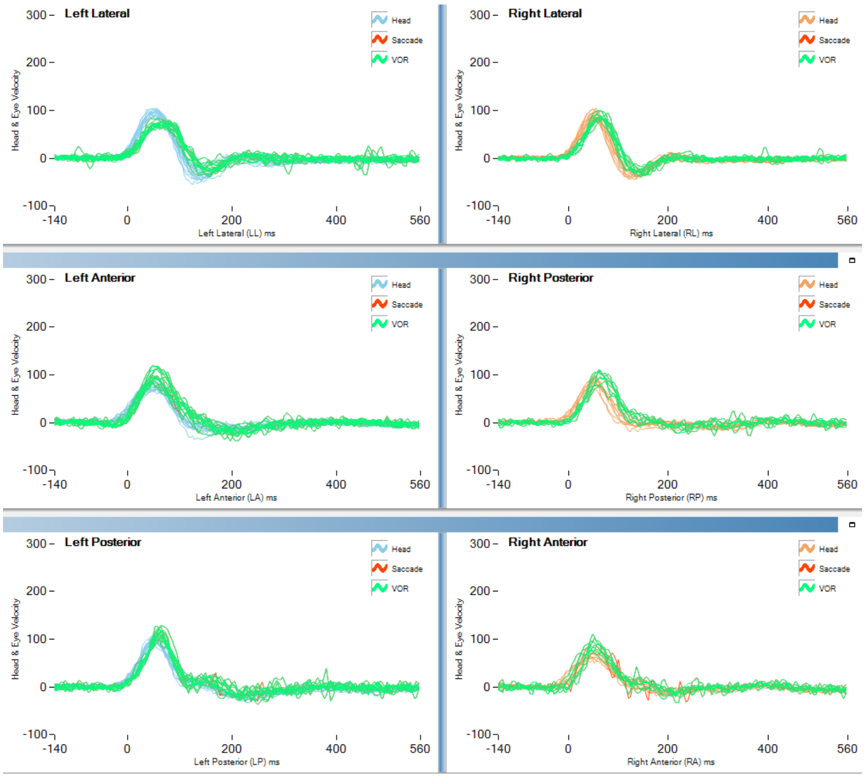

Figure A8: vHIT traces of the second test (>300h & <2000h, aerobatic, no F/A 18) of pilot 3 of study 2

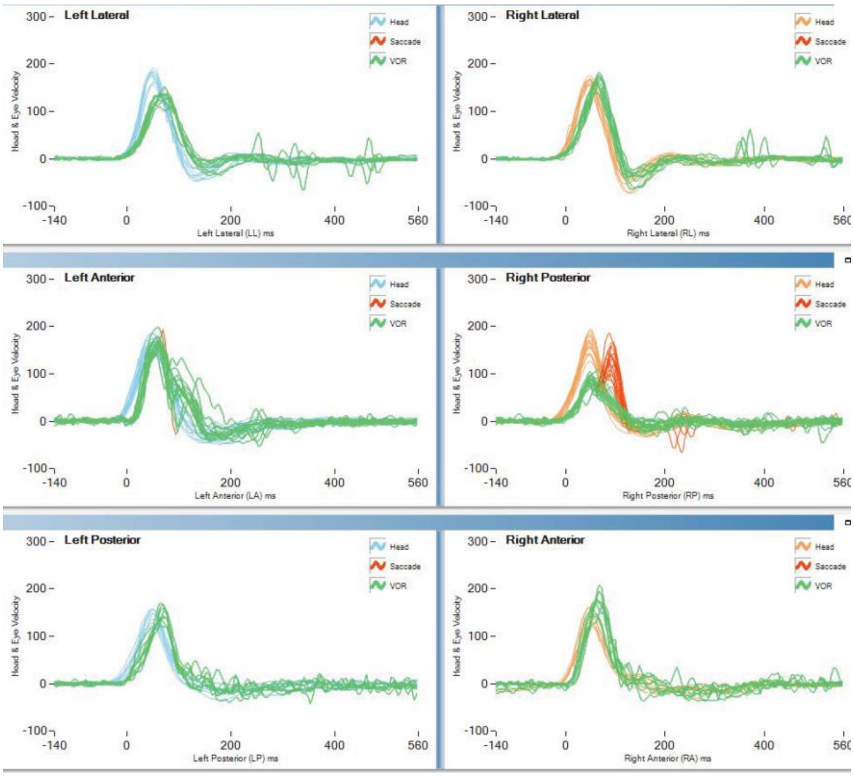

Figure A9: vHIT traces of the last test (>2000h, aerobatic & F/A 18) of pilot 3 of study 2

<u>**Pilot 4: Violet line in Figure 1**</u>

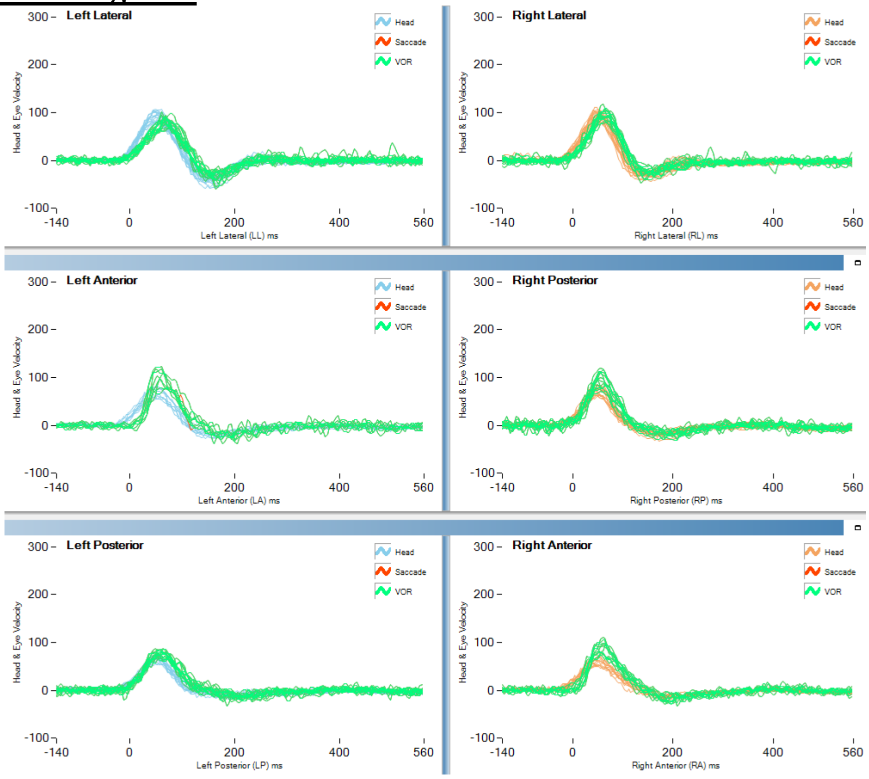

Figure A10: vHIT traces of the first test (<300h no aerobatic, no F/A 18) of pilot 4 of study 2

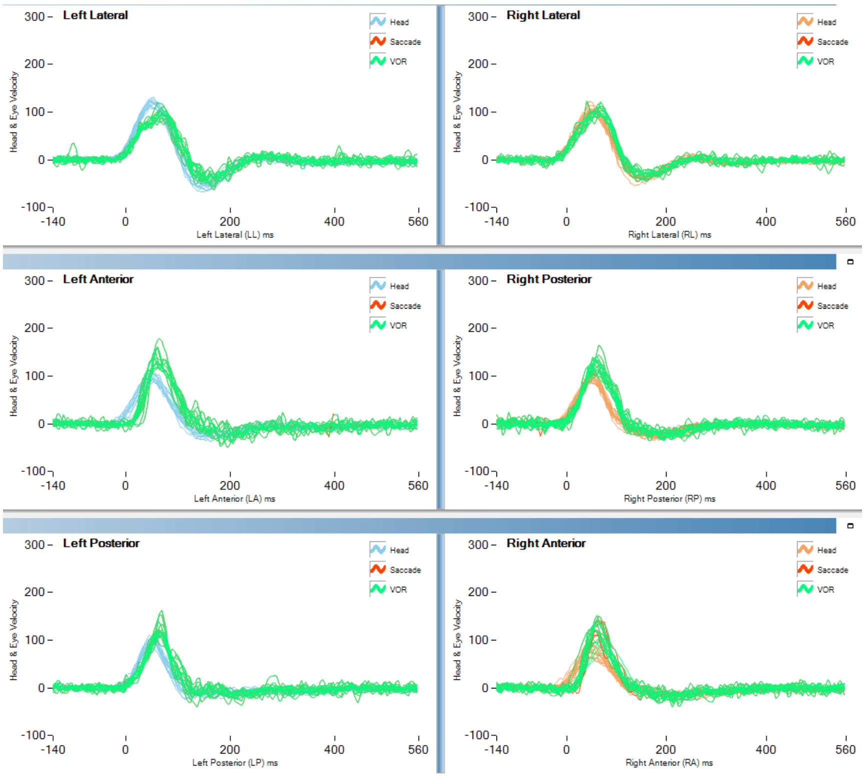

Figure A11: vHIT traces of the second test (>300h & <2000 h, aerobatic, no F/A 18) of pilot 4 of study 2

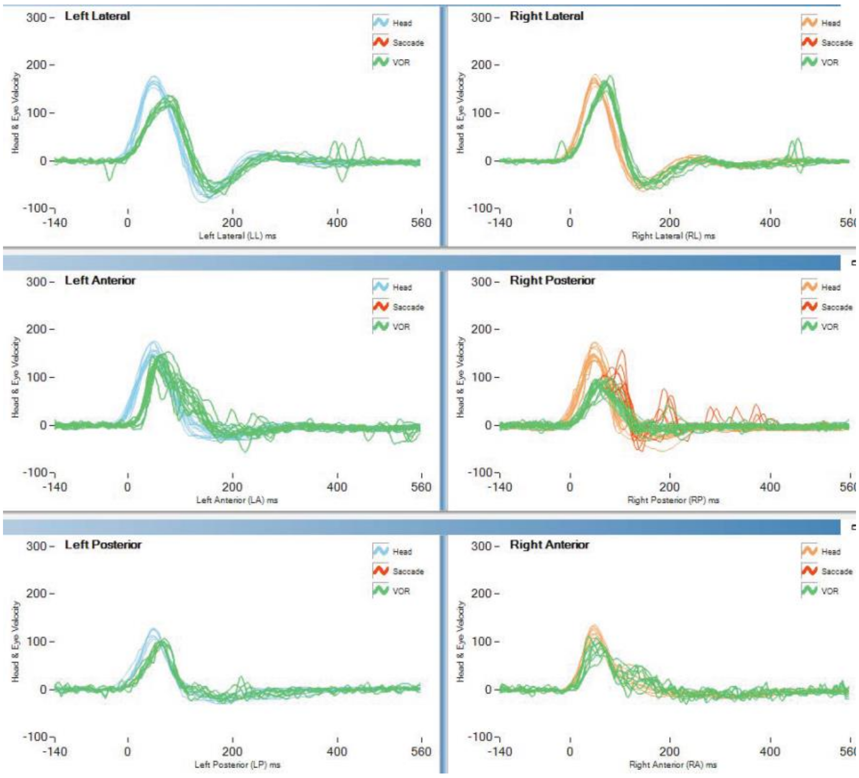

Figure A12: vHIT traces of the last test (>2000h, aerobatic & F/A 18) of pilot 4 of study 2
